# 3D printed skulls in court — a benefit to stakeholders?

**DOI:** 10.1007/s00414-023-03054-6

**Published:** 2023-07-01

**Authors:** Mikkel Jon Henningsen, Lykke Thorlacius-Ussing, Lotte Groth Jensen, Kasper Hansen, Christina Jacobsen, Stina Lou, Chiara Villa

**Affiliations:** 1 Section of Forensic Pathology, Department of Forensic Medicine, Faculty of Health and Medical Sciences, University of Copenhagen, Copenhagen, Denmark; 2 DEFACTUM, Public Health & Health Services Research, Central Denmark Region, Aarhus, Denmark; 3 Department of Forensic Medicine, Faculty of Health, Aarhus University, Aarhus, Denmark

**Keywords:** 3D print, Post-mortem computed tomography, Interview study, Skull, Court of law

## Abstract

Forensic pathologists may use 3D prints as demonstrative aids when providing expert testimony in court of law, but the effects remain unclear despite many assumed benefits. In this qualitative study, the effects of using a 3D print, demonstrating a blunt force skull fracture, in court were explored by thematic analysis of interviews with judges, prosecutors, defence counsels, and forensic pathologists with the aim of improving the expert testimony. Five semi-structured focus groups and eight one-to-one interviews with a total of 29 stakeholders were transcribed *ad verbatim* and analysed using thematic analysis. The study found that a highly accurate 3D print of a skull demonstrated autopsy findings in detail and provided a quick overview, but sense of touch was of little benefit as the 3D print had different material characteristics than the human skull. Virtual 3D models were expected to provide all the benefits of 3D prints, be less emotionally confronting, and be logistically feasible. Both 3D prints and virtual 3D models were expected to be less emotionally confronting than autopsy photos. Regardless of fidelity, an expert witness was necessary to translate technical language and explain autopsy findings, and low-fidelity models may be equally suited as demonstrative aids. The court infrequently challenged the expert witnesses’ conclusions and, therefore, rarely had a need for viewing autopsy findings in detail, therefore rarely needing a 3D print.

**Supplementary Information:**

The online version contains supplementary material available at 10.1007/s00414-023-03054-6.

## Introduction

The first 3D print of a human skull based on computed tomography (CT) data was made more than 30 years ago [1]. 3D prints of human skulls have been used as demonstrative aids in court of law since 2009 [2], and 3D prints are now commonly used in English and Welsh courts [3]. However, much is assumed about the benefits of using 3D print in court, some is inferred, and little is known. The Swiss Virtopsy project suggested in a proof-of-concept paper that 3D printing overcomes the loss of information from viewing 3D volume renderings on 2D screens and provides a haptic component “necessary for educational purposes and in presentations to medical laymen” [4]. Kettner et al. argued that 3D prints have the benefit of showing bone fragments in situ, i.e. not “fallen out” as may happen during autopsy [2]. Baier et al. stated the additional benefits of no health hazard to jurors and no risk of damage to human tissue [5]. The potential for subconscious bias from emotional unease against the defendant was the only expected drawback [5]. All three papers suggest that 3D printing is less emotionally disturbing to judges, lawyers, and relatives than photographs when presenting autopsy findings in court [2, 4, 5].

A study on the effects of different evidence formats found no difference between 3D print and photos for “evidence complexity”; however, jurors felt 3D print improved their “understanding” [6]. A more recent study did not demonstrate any benefits of 3D prints [7]. Explaining autopsy findings to jurors may be compared to teaching, and the effects of 3D prints in teaching anatomy to medical students are ambiguous [8–12].

The objective of this study was to explore the effects of using a 3D print demonstrating blunt force skull fracture in court by means of interviews with relevant stakeholders and thematic analysis of their responses.

## The Danish legal system in brief

In Danish criminal law, the Prosecution Service is part of the police and is bound to present exculpatory evidence. In court, the defence counsel may also present evidence but is not bound to disclose incriminating evidence omitted by the prosecution. Both parties may bring any evidence, and the prosecution and defence counsel usually agree on what evidence to bring forward with the court deciding in cases of disagreement. The court has free assessment of evidence, meaning that it may attribute whatever weight it decides to any evidence. In Danish courts, jurors participate with equal rank and responsibility as the legal judge(s), deciding on both guilt and length of sentence [13].

## Methods

Several recruitment strategies for interview participants were employed, including advertising at conferences, using professional mailing lists, and personal networks. Once recruitment had begun, snowballing was also used. We aimed for a sample of maximal variation based on profession, seeking prosecutors, defence counsels, judges, and forensic pathologists. To provide for a solid and trustworthy analysis, recruitment continued until sufficient information power was reached [14]. Due to the record-keeping procedures of the Danish Courts and the Danish Data Protection Act, it was impossible to identify jurors who had participated in criminal trials with forensic pathologists as expert witnesses.

The interview guide was iteratively developed based on the research question, existing literature, and informal interviews with relevant stakeholders, and piloted. Interviews were in Danish, but an English translation of the interview guide is available in the supplementary materials.

To promote discussions of 3D printed evidence during the interviews, a suitable case with several linear fractures of the skull and a trauma mechanism with two impacts was identified. Circumstances such as date, location, age of the deceased, non-relevant autopsy findings, and details of the event were changed to anonymise the case. Head injuries were kept consistent with the actual case. The case imitated a standard autopsy report and was given to the participants prior to introducing the 3D print in the interview situation.

**Fig. 1:**
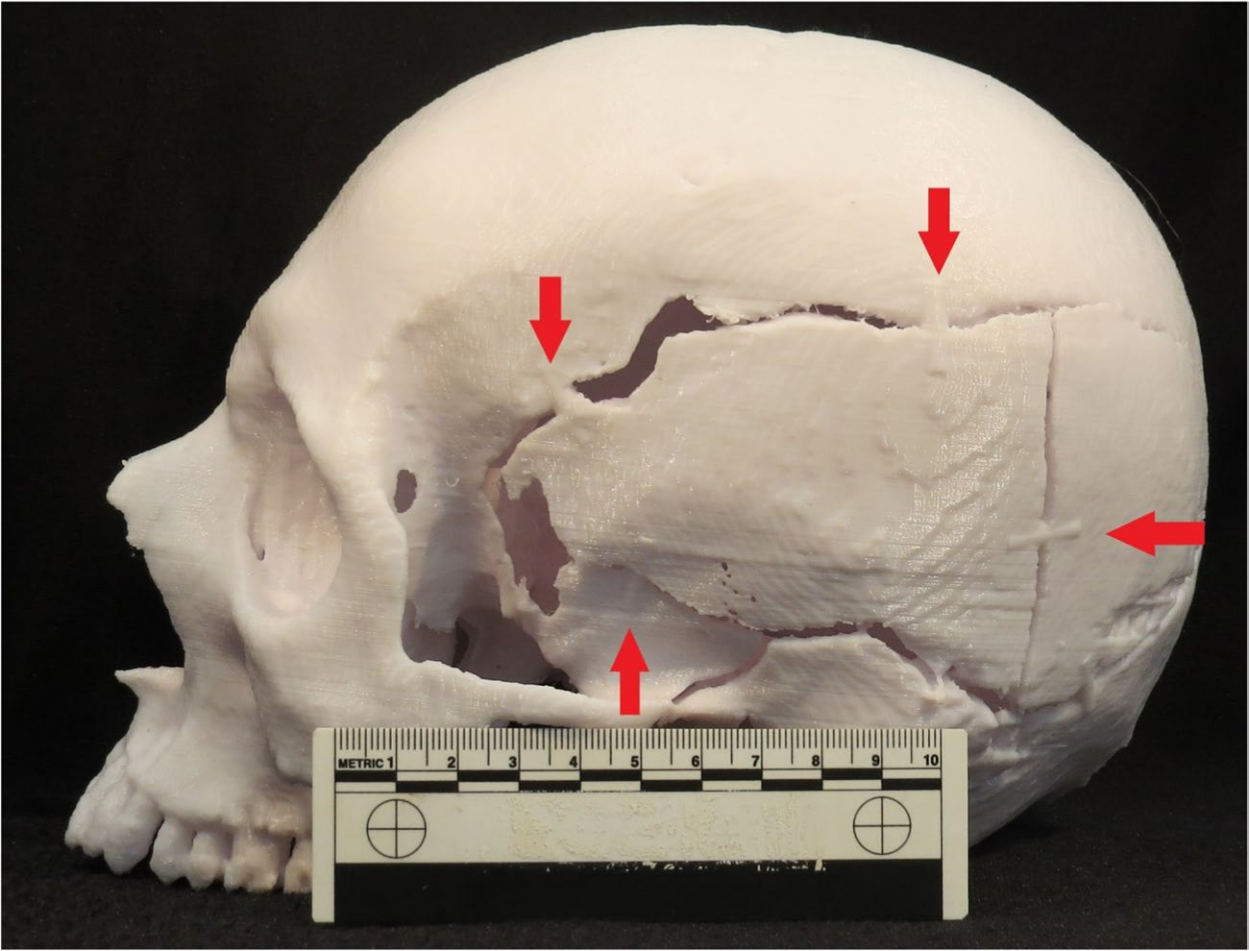
Photo of the 3D print seen from the left with inserted rods marked (red arrows)

The 3D print was based on CT data obtained using a clinical CT system (64-slice Siemens Somatom Definition, Siemens Medical Solutions, Forchheim, Germany) with the following settings: 120 kVp, dose modulated tube current of 342 to 630 mA, rotation time 0.5 s, field of view 500 mm, slice thickness of 0.75 mm at 0.6 mm increments, pitch 0.80, and reconstructed with sharp algorithm (h60f). Segmentation was performed with 3D Slicer v. 4.11 [15] using tools in the “segmentation editor”-module, including “thresholding”, and manual correction of artefacts (small holes) with “paint” and “erase” tools. For better alignment with the case story, the fracture lines and sutures were “enhanced” locally. To keep bone fragments from dislodging in the physical model, “Blender” (v. 3.2) [16] was used to insert cylindrical rods acting as support for the loose structures (red arrows in image 1). Printing was performed using an Original Prusa i3 mk. 3 (Prusa Research a.s., Prague, Czeck Republic) with a layer height of 0.2 mm and took 12 h (material costs; 3£). The finished 3D printed model is shown in Fig. 1.

Focus groups and individual interviews were conducted by MJH at different public or private meeting facilities. LTU participated in group interviews to aid note keeping, time management, observations of body language, use of irony, and topics to explore further. All interviews were digitally recorded, transcribed *ad verbatim*, and rendered anonymous.

**Fig. 2:**
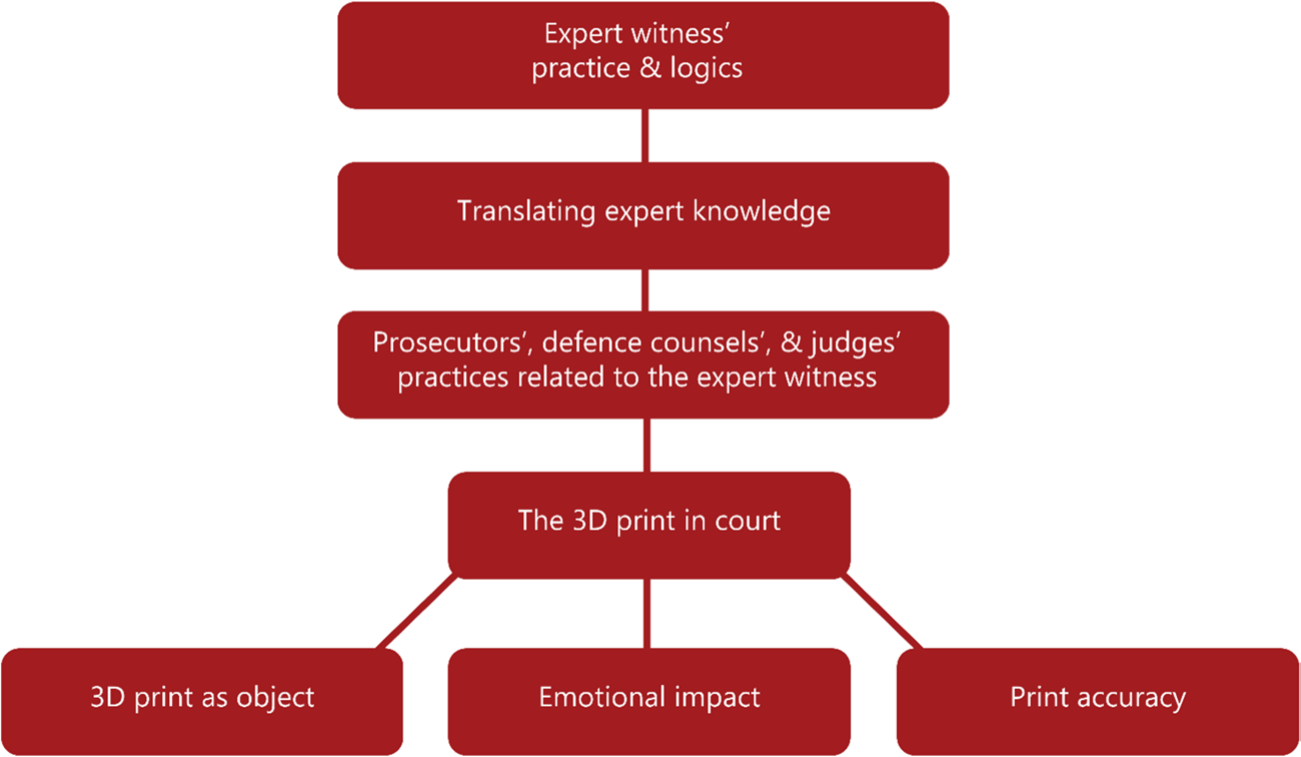
Thematic map

We used a pragmatic version of reflexive thematic analysis, as thoroughly described by Braun et al. [17]. First, interviews were read in their entirety. Then, a coding guide was constructed based on the initial impressions, study objectives, assumptions about stakeholders’ roles, and the effect of different evidence/testimony modalities. This guide, available in the supplementary materials, was used for deductive coding of the interviews to roughly sort the material and make the initial meaning of it. After this first round of coding, all statements in the interviews that appeared important but did not fit any of the pre-established codes were assigned a new code based on the statements’ content. Interviews and coding were managed with NVivo 12 (QSR International Pty Ltd., Melbourne, Australia). The themes were iteratively illustrated in thematic maps and then revised, resulting in the final thematic map presented in Fig. 2.

## Results

As summarised in Table 1, for a total of 29 respondents, five were focus group interviews and eight were 1:1 interviews. Interviews lasted from 48 to 110 min.

**Table 1:** The respondents’ profession, number of participants present, and duration of interviews

Respondent(s)	Participants	Duration (minutes)
Prosecutors	5	80
Defence counsel	1	62
Defence counsel	1	49
Prosecutor	1	57
Defence counsels	6	100
Defence counsel	1	59
Prosecutor	1	98
Judges	5	110
Prosecutor	1	68
Forensic pathologist	1	48
Forensic pathologists	3	87
Forensic pathologists	2	83
Forensic pathologist	1	62

When analysing data, we initially created partly interconnected themes. After discussion, we decided that the most relevant major themes to our objective were the “translating expert knowledge” and “the 3D print in court”. Accordingly, a description of the major and minor themes is visible in the “thematic map” (Fig. 2).

### Theme 1: Translating expert knowledge

The first major theme describes the stakeholders’ perceptions of the expert witness and their need for translation of the forensic pathology findings. This is a necessary first step in understanding the effects of 3D prints, as these perceptions shape how stakeholders view, understand, and use forensic information and data in court.

#### Subtheme 1.1: Expert witnesses’ practice and logics

The written autopsy report was perceived by all professions as objective, unchallengeable, and a tool for establishing common ground for debating guilt and intent by establishing the outcome of the alleged events. Despite the forensic pathologists’ emphasis on writing the report in non-technical language, all prosecutors, defence counsels, and judges found the written report difficult to understand. Therefore, in court, they needed the forensic pathologist to translate it into a contextual language, making autopsy findings understandable in the specific case they argued.

Judge G5: *“I would certainly need an explanation for “occipital bone”, “from left towards centre line”, I mean “vault”, “parietal bone”… Where is that?”*

The forensic pathologists considered it their role to document autopsy findings, communicate concisely, and translate technical language. They were cautious about neither speaking beyond their competencies nor generating hypotheses. Forensic pathologists described their approach as “reactive” as they would answer questions concisely, not elaborate on their own accord, and be taciturn. However, pathologists would sometimes volunteer information or elaborate if the defence counsel blatantly failed to ask relevant questions, presumably to prevent a perceived miscarriage of justice.

Forensic pathologist G1: *“Answer the question you are asked! […] And then you can be itching to say something or be annoyed that the prosecutor did not phrase the question differently, but that is the legal process, and we should not interfere with that.”*

#### Subtheme 1.2: Prosecutors’, defences counsels’, and judges’ practices and logics related to the expert witness

Prosecutors and defence counsels considered it their responsibility to make sure all relevant facts and reservations were understood by judges and jurors. For this purpose, they needed the expert witness to translate and elaborate on the forensic findings.

The respect for the forensic pathologists was significant, and their statements carried great weight in court. Prosecutors, judges, and defence counsels accepted these statements with little (if any) reservation, because they themselves lacked the medical expertise to draw conclusions from autopsy findings. Prosecutors and defence counsels found it difficult to ask the forensic pathologists questions that elicited the information they needed and valued experts who would explain on their own initiative. Defence counsels experienced the forensic pathologists’ reactions to their trouble with phrasing questions as scepticism towards them. Judges, prosecutors, and defence counsels relied on the expert for explaining not only anatomy and objective findings, but also the meaning of these findings, and whether they were in accordance with proposed events. Prosecutors and defence counsels rarely requested elaborations on specific details and rarely wanted to see, e.g. fractures themselves, but preferred to rely on the experts’ conclusions. Defence counsels found the latter problematic as they considered it the judges’ and jurors’ prerogative, and not the forensic experts’ task, to reach a conclusion based on the facts presented in court, i.e. assessing evidence.

Defence counsel G5: *“Perhaps we need to be better at getting help to utilise the experts correctly.”*

All participants expressed a strong professional ideal of treating the victims respectfully and being mindful of the potential emotional impact of the expert witness statement on judges, jurors, and relatives. All recognised the need for information but also adhered to the principle of using the least possible to achieve the sufficient. For example, if photos contributed with something that could not be learned by other less unpleasant means, then the emotional impact was acceptable.

While prosecutors and defence counsels used the expert witnesses to convey facts, prosecutors also reported to use the expert witness to appeal to the emotions of judges and jurors and/or to imply intent. Prosecutors requested firm conclusions in clear language, yet recognised that potential reservations from the expert witness served to uphold his/her trustworthiness. The experts’ hesitance to use definitive language sometimes frustrated prosecutors when upholding reservations which the prosecutors considered “theoretical doubt”. By “theoretical doubt”, prosecutors meant scientific reservations that were possible but not plausible and, in their mind, mistakenly perceived by judges and jurors as the legal concept of “reasonable doubt”. The defence counsels considered it their role to explore alternatives to the prosecution’s hypotheses. In their encounter with the expert witness, they therefore sought to test the plausibility of alternative hypotheses, to maximise doubt (including “theoretical doubt”), and/or to challenge the expert witnesses on wording.

### Theme 2: The 3D print in court

This theme elaborates on the courts’ need for explanatory aids and focusses on the perceived benefits and challenges when using 3D prints. From our interviews, it was apparent that the 3D print as a physical object, the model fidelity, and the emotional impact were intertwined. The 3D-printed skull differed from currently used aids by the combination of being a physical object and visually accurate.

#### Subtheme 2.1: 3D print as an object

All professions stated that the 3D print looked real and felt fake. Participants used their hands to orient the 3D print of the skull for visual inspection, but extracted little to no information by touch itself, thus benefitting little from the “haptic component”. We observed the ease with which stakeholders handled the skull, imitated movements, and spontaneously pointed to the lesion or anatomy they were enquiring about, thus compensating for the lack of medical vocabulary. Compared to photos, the 3D print clearly enabled change in viewpoint and movement. Because the 3D print had a different weight and strength than a human skull, sense of touch was misleading or irrelevant, as illustrated by the quote below. Jurors lifting a heavy bat would know the intent needed to swing it, but jurors lifting a 3D-printed skull would not know how strong a real skull was.

Defence counsel 2: *“This 3D print is different to a heavy bat, because with the bat, a prosecutor could say “Feel how heavy this is, it weighs 20 kilos. If I am hit in the head with this, what do you think would happen?” But here, with this 3D print, it makes no odds.”*

In several interviews, it was noted how the 3D print would be difficult to see from a distance, unlike sketches or photos that may be projected on screens. The forensic pathologists also argued that judges and jurors would see the 3D print from different angles while hearing the forensic pathologist’s explanation, which they perceived as potentially confusing.

Some defence counsels pointed out that pre-court review and post-court archiving of 3D-printed case material would be cumbersome compared to digital material. In most interviews, the physical 3D print was spontaneously compared with virtual 3D models, which were perceived as logistically easier, less emotionally confronting, and equally suited for explaining fractures. Only the forensic pathologists preferred 3D prints to potentially unfamiliar software.

Like sketches and overview photos, the 3D prints were expected by all participants to enable an impression of lesions, which they could not achieve by reading the autopsy reports.

Forensic pathologist G4: *“You get an instant overview of what has happened to the skull. When you read an autopsy report, you must visualise it inside your own head. With the 3D print you are given this visualisation, which is a significant advantage.”*

Sketches and photos could sometimes be confusing due to a mix of old and new injuries, whereas 3D prints could be confusing due to the many details. Defence counsels pointed out that 3D prints could benefit their cause in cases with avulsions or hairline fractures as the word “fracture” was usually understood as a complete break and often associated with displacement. This “calibration” of meaning was also possible with photos or X-ray images; however, these were perceived as more difficult for laypersons to interpret.

#### Subtheme 2.2: 3D print accuracy

When asked about their attitudes towards the accuracy of the 3D print, all professions agreed that high fidelity added credibility to the 3D print and to the forensic pathologist. Prosecutors were concerned that inaccurate 3D prints would be a point of attack for defence counsels. However, the defence counsels stated this would be an inefficient strategy, as the autopsy report, not the 3D print, was the authoritative documentation. Among the forensic pathologists, some refused to use anything but exact 3D prints for fear of losing credibility, while others argued that simple props, such as an egg with line drawings, could effectively provide the benefits of three-dimensional demonstration. Most forensic pathologists agreed that too many details were distracting to medical laypersons.

Like the use of photos and radiological visualisations in court, forensic pathologists would have to explain the 3D print regardless of accuracy for laypersons to understand autopsy findings and implications thereof. A high level of detail was not needed by the court, as prosecutors, defence counsels, and judges had no desire to view specific anatomical details, which was also reflected in the practice of “believing” the experts’ statements regarding organ injury rather than wanting to view the autopsy photos themselves. All professions agreed that errors in the 3D print were acceptable on the condition that they were clearly marked and not influencing the perceived severity of the lesions.

Prosecutor 2: *“I do not think it has any relevance where exactly the fracture lines run.”*

#### Subtheme 2.3: Emotional impact of 3D print

All participants were concerned about the potential emotional burden on living victims, relatives, and jurors during a trial. Several prosecutors noted that the absence of blood and soft tissue on 3D prints could be less emotionally confronting than photos; however, photos remained necessary for demonstrating soft tissue injury.

We observed an interesting disparity as some participants, indifferent of their professions, found the 3D print emotionally unremarkable, often comparing it to toys or skulls seen in museums, whereas other participants explicitly stated that they found the 3D print emotionally confronting. The prosecutors and defence counsels who did not find the 3D print emotionally disturbing themselves still believed that the 3D print appealed to the emotions of jurors and judges.

For both 3D prints and gruesome photos, prosecutors were concerned that jurors and judges would look away or get emotionally affected, which would affect their ability to objectively evaluate and remember information. However, prosecutors argued that unpleasant photos could also be intentionally used to arouse emotions to strengthen the prosecutors’ position. Other prosecutors and defence counsels argued that gruesome photos were a necessary evil to document lesions and should be avoided if other media, such as 3D prints, were sufficient.

Prosecutor G2: *“I would rather look at the 3D print than at autopsy photos. The 3D print, on the table there, it does not bother me, but had it been the photo-folder, then I would have closed it.”*

Prosecutor 1: *“A super [autopsy] photo with probes demonstrating the wound canal, and then this other prosecutor said to me “This photo is too gruesome, I cannot show this in court.” It is the primary evidence! No one who sees the depth of this wound will doubt that the intention was to kill, so of course you must show the photo.”*

Most participants perceived the skull fractures and the events that had caused them as more severe after seeing the 3D print compared to only reading the autopsy report. However, most had no perception of the skull fractures from the autopsy report. This change in perception of severity was not experienced as problematic, as all professions considered it a result of more information and something that other types of visual aids, such as sketches and photos, could also have achieved.

It was noted that errors reminded the respondents that the 3D print was a copy, which was perceived as an advantage because it lessened the emotional impact. By extension, some considered highly accurate 3D prints ethically troublesome as they believed that detailed 3D prints raised the emotional impact compared to a standardised skull. Like errors, the use of colours and a print smaller than 1:1 scale were suggested by prosecutors, defence counsels, and forensic pathologists to lessen the emotional impact of 3D prints. All professions suggested that a virtual 3D model would accomplish the same as a physical 3D print but might lessen the emotional impact even more.

## Discussion

The objective of this study was to explore the effects of introducing 3D prints in court as demonstrative aids to the forensic pathologists’ expert witness testimony. 3D prints of human skulls have been used both in civil law and case law [2, 5]. The rules of evidence admissibility and practice of using either court-appointed experts or adversarial experts vary, but it is reasonable to assume that the effects of 3D prints and the role of the expert witness are comparable across legal traditions.

This study and the literature demonstrated that judges and jurors found it difficult to comprehend technical language and statistics [18–21] and that they appreciated expert witnesses who used visual aids and translated technical language [22], as visual aids increased jurors’ perceived evidence understanding [6]. All participants considered the expert witnesses very credible, which resonates with prior research [22], and prosecutors, defence counsels, and judges relied on the expert witnesses for assessing the plausibility of proposed scenarios, underscoring the important role of expert witnesses as translators of specialised knowledge. They also accepted the expert witnesses’ statements and conclusion almost without reservation, also previously demonstrated [23] and criticised [24]. In line with prior research, prosecutors and judges in the current study preferred firm statements from the expert witnesses [25], whereas defence counsels both needed firm statements but also benefitted from hedged statements to create doubt. Expert witnesses use of hedging has previously been documented in a discursive analysis of trial transcripts [26]. The current study demonstrated a distinct discrepancy between especially the prosecutors’ and judges’ need for firm statements and the expert witnesses’ hedged answers. The differences in phrasing between medicine and law have previously been demonstrated as problematic [27]; however, defence counsels stated that they could also benefit from this difference.

The ability to touch, as opposed to only see, has previously been speculated to improve juror understanding of autopsy findings [4]. However, respondents in this study generally rejected the notion that touch added additional information and the “haptic component” was thus perceived to have marginal benefit. The primary benefit of touching a physical 3D model was the ease with which it was positioned for visual inspection. Our results also point to how physical 3D prints present logistical challenges in pre-trial distribution, in-trial demonstration, and post-trial archiving. The possibility to digitally send fragile or invaluable objects such as archaeological specimens and then print locally has previously been presented as an advantage of 3D printing [28], and defence counsels in this study also approved of only receiving a virtual 3D model prior to trial. However, increasingly digital societies are not designed for physical objects such as 3D prints in terms of transport, presentation, and storage. No prior studies have addressed these logistical issues besides noting the benefit of digital storage and *ad hoc* printing when the object is broken or needed. We speculate that these logistical challenges could be a barrier to routinely using 3D prints in court. Destruction of the 3D print after use and storage of only the 3D print digital file may lessen this challenge. Photos of 3D print and the physical 3D print have previously been demonstrated to perform equally well for jurors’ perceived evidence understanding [6], though autopsy photos and 3D prints were equally good and better than photos of 3D prints for reducing the perceived complexity of expert witness testimony [6]. Other studies have also demonstrated photos, 3D prints, and photos of 3D prints to perform equally for self-rated perception of understanding and complexity of evidence [7]. In one study, all formats were demonstrated on video thus negating touch [6], but participants were able to touch in the other study [7]. The preference for virtual models was also voiced by participants in this study [7].

In studies on 3D print accuracy, high-fidelity is implicitly assumed beneficial [29, 30], reflecting the common notion that higher fidelity is better [31]. Research on cognition and information extraction from visual displays have indicated that low-fidelity is more beneficial [31]. The reason is speculated to be that high-fidelity displays place the burden of perceiving, prioritising, and evaluating on the user in contrast to simplified displays. Simplified displays make perception easier by reducing “noise”, make prioritisation easier by removing irrelevant information, and thus reduce the cognitive burden for evaluation [31]. Respondents who discussed details as confusing or distracting may have expressed this without a theoretical framework. Among the forensic pathologists in our study, it was debated whether high-fidelity or low-fidelity 3D prints were best suited for conveying autopsy findings, whereas the prosecutors, defence counsels, and judges voiced that high-fidelity 3D prints were very credible and could enhance the expert witnesses’ credibility. Forensic pathologists already used low-fidelity visual aids such as scrunchies (explaining the hymen) with good effect. Similarly, other research suggests that low-fidelity models are best suited for medical novices [32], which jurors and judges may be considered to be. Non-medical respondents in this study expressed the need for an expert to explain even the high-fidelity 3D print, and that a simplified model would be sufficient to meet the demands of the court in most cases, i.e. was the victim struck on the right or left side of the head, rather than was the victim struck 2 mm or 3 mm above the suture. However, our results provided no clear answer to these debates. The norms of forensic pathologists may dictate high-fidelity documentation, but the needs of other stakeholders may warrant low-fidelity prints. Future studies may compare low-fidelity with high-fidelity models for juror understanding. Nevertheless, our findings suggest that regardless of the fidelity, a thorough explanation of the 3D print by the forensic pathologist is necessary.

In our analysis, 3D prints were considered by most respondents to be emotionally disturbing to jurors regardless of emotional impact on themselves. Blau et al. found that “legal professionals” deemed photos less confronting than other professions [6], thus our study might underestimate the emotional impact of 3D prints. Respondents spontaneously compared 3D prints with photographs and assumed that 3D prints were less emotionally disturbing, arguing that when 3D prints could replace photographs, they should. Indeed, prior studies have found 3D prints to be less emotionally disturbing than autopsy photos, though still more emotionally disturbing than photos of 3D prints [6]. Respondents in this study speculated that virtual 3D models would be less emotionally confronting than physical 3D prints. It is well known that emotionally confronting evidence bias towards conviction [33–38], and the more complex the subject of the expert witness testimony and the less the jurors understand, the more they rely on heuristics [39]. However, the issue of emotional impact when 3D printing human anatomy needs further investigation.

The absence of jurors poses a limitation in that they may react emotionally different to the 3D print than the participating stakeholders. The composition of the focus groups may mitigate this somewhat in that both a newly qualified prosecutor and defence counsel participated in the respective interviews. Judges provided insight on juror behaviour and reasoning, and studies indicate that judges and jurors have similar understanding of scientific evidence [40]. Another limitation is selection bias in that those participating may be more open-minded to 3D print than those declining participation [41].

This study had several strengths: the study was able to sample all relevant professions, used a 3D print of a skull subjected to blunt force trauma and a forensic case for discussion, performed concurrent transcription of interviews allowing assessment of information power, and used researcher triangulation throughout data collection and analysis to enhance trustworthiness of results.

## Conclusion

The high-fidelity 3D print of a fractured skull demonstrated the autopsy findings in detail to non-medical stakeholders thus meeting the forensic pathologists’ professional norm of thorough documentation, yet remained superfluous as the court was less interested in seeing the reasoning behind the forensic pathologist’s conclusion, as they accepted the conclusion with little reservation. The physical 3D print was perceived to have few benefits compared with virtual 3D models as sense of touch provided little additional information to vision. Additionally, virtual 3D models were speculated to be less emotionally disturbing than 3D prints. Both physical and virtual 3D models were expected to be less emotionally disturbing than photos.

Future research could investigate the emotional and educational benefits of highly accurate 3D prints vs. “toy skulls” with fractures drawn on them, by administering tests on comprehension, knowledge recall, and emotional state with, e.g. the POMS-40 questionnaire.

### Supplementary information

ESM 1

ESM 2

## Data Availability

The dataset (interviews) generated during during the current study are not publicly available as participants only consented to external data sharing in anonymized form. Since full transcripts cannot be fully anonymized due to the highly individual context, the transcripts can only be made available upon reasonable request and special conditions may apply. Any requests concerning data access can be directed to the corresponding author.
