# Supplementary material for: 3D printed skulls in court — a benefit to stakeholders?
Source: Int J Legal Med. 2023 Jul 1;137(6):1865–73. doi: 10.1007/s00414-023-03054-6 (PMC10567900; doi:10.1007/s00414-023-03054-6)
Supplement: Supplementary file 1 — ESM 1 [file 414_2023_3054_MOESM1_ESM.docx]

| **Question** | **Question** | **Probes** |
| --- | --- | --- |
| # 1 | Would you briefly describe your background |  |
| # 2 | In a trial, how do you perceive the role of the forensic pathologist’ expert witness statement? | Compare / contrast with other witnesses, other “evidence” |
| # 3 | What is important to you, regarding the expert witness statement? | To elucidate  Relating to your objectives in court |
| # 4 | How would you describe a very usable expert witness and expert witness statement? | What made it ”good”?  What is the “ideal” expert witness statement? |
| # 5 | How would you describe a less usable expert witness and expert witness statement? | What made it ”bad”? |
| # 5.5 | Have you experienced expert witnesses’ (statements) that were special / peculiar / made a difference / unusual? | How so? |
| # 6 | How do you use the expert witness statement? | How is it a help / hindrance to you and your objectives? |
|  | Hypothetical autopsy report handed to everyone.  3D print introduced when done reading |  |
| # 7 | When you see this 3D print, how does it compare with your impressions from the autopsy report? |  |
| # 8 | What do you see on the 3D print? | Where do you see it?  What anatomical features do you recognize?  Pathologists only: What do you think of the anatomical quality? |
| # 9 | What does the 3D print feel like?  *(Ambiguous question in Danish, “feel” may both indicate emotions evoked and concrete, tactile feel)* | Concrete: Heavy/light, solid/fragile, plastic-like, confident in handling  Emotionally  To use in trial  If no emotional response:  ”This is a 1:1 copy of a real person’s skull – what is that like to know? |
| # 10 | What could have caused what you see on this 3D print? | Event, force, direction, severity  Artifacts, manipulation |
| # 11 | Does the 3D print change you perception of the defendant / victim? | Would you argue for a different punishment? |
| # 12 | Would you like forensic pathologists to use 3D prints as demonstrative evidence in court? | Why (not)?  How would you prefer it being used? |
| # 13 | What might 3D prints be suitable for? |  |
| # 14 | What might 3D prints be less suitable for? |  |
| # 15 | What problems do you imagine that the use of 3D prints could cause? |  |
| # 16 | What would the use of 3D prints do to the forensic pathologists’ expert witness statement |  |
| # 17 | How would 3D prints effect you in reaching your objectives in court? |  |
| # 18 | Is there anything you would like to discuss or elaborate on? |  |
