# Supplementary material for: 3D printed skulls in court — a benefit to stakeholders?
Source: Int J Legal Med. 2023 Jul 1;137(6):1865–73. doi: 10.1007/s00414-023-03054-6 (PMC10567900; doi:10.1007/s00414-023-03054-6)
Supplement: Supplementary file 2 — ESM 2 [file 414_2023_3054_MOESM2_ESM.docx]

Deductive coding structure

| **Material** |  | Written – verbal – visually (including 3D) - objects | |
| --- | --- | --- | --- |
|  | Verbal | Statements regarding this ”material” | Strictly about material. If pertaining to the expert witness statement, then code as such |
|  | Written | Do | Do |
|  | Visual | Do | Do |
|  | Physical objects | Statements regarding this ”material” – 3D prints, knives, models, etc. | Do |
| **Roles** |  |  |  |
|  | Defence counsels – professional logic | Expectations, norms, ethics, language of own profession | Also about ”information” vs. ”drama” effect of evidence |
|  | Prosecutors –  professional logic | Do | Do |
|  | Judges –  professional logic | Do | Do |
|  | Forensic pathologists –  professional logic | Do | Do |
|  | Expert witness statement | What expert witnesses can, should, do, and may be used for.  Also expectations from other stakeholders.  About all materials that are part of expert witness statement | Expected to be large – sub-codes inductively |
|  | ”The court” | What judges and jurors (and prosecutors and defence counsels) can, do, should, and are used for.  Also other’s expectations.  Also ”**strategies for understanding**” | Including “evidence assessment”, “evidence evaluation” and who may do what (pertaining to roles) |
| **3D print** |  |  |  |
|  | ”Fidelity” | Speculations about ”correctness” and printing precision |  |
|  | Expectations and assumptions regarding the use of 3D print  Also “wishes” and “demands” for using 3D print | Speculations on practical, theoretical, philosophical consequences of using 3D print.  Including advantages, drawbacks | Expected to be large – sub-codes inductively |
